# Effect of temporal sampling protocols on myocardial blood flow measurements using Rubidium-82 PET

**DOI:** 10.1007/s12350-021-02555-4

**Published:** 2021-03-02

**Authors:** S. S. Koenders, J. D. van Dijk, P. L. Jager, M. Mouden, A. G. Tegelaar, C. H. Slump, J. A. van Dalen

**Affiliations:** 1 Department of Nuclear Medicine, Isala Hospital, PO Box 10400, 8000 GK Zwolle, The Netherlands; 2 Department of Medical Physics, Isala hospital, Zwolle, The Netherlands; 3 Department of Cardiology, Isala hospital, Zwolle, The Netherlands; 4 Technical Medical Center, University of Twente, Enschede, The Netherlands

**Keywords:** Myocardial blood flow, PET myocardial perfusion imaging, ^82^rb, temporal sampling, regadenoson

## Abstract

**Background:**

A variety of temporal sampling protocols is used worldwide to measure myocardial blood flow (MBF). Both the length and number of time frames in these protocols may alter MBF and myocardial flow reserve (MFR) measurements. We aimed to assess the effect of different clinically used temporal sampling protocols on MBF and MFR quantification in Rubidium-82 (Rb-82) PET imaging.

**Methods:**

We retrospectively included 20 patients referred for myocardial perfusion imaging using Rb-82 PET. A literature search was performed to identify appropriate sampling protocols. PET data were reconstructed using 14 selected temporal sampling protocols with time frames of 5-10 seconds in the first-pass phase and 30-120 seconds in the tissue phase. Rest and stress MBF and MFR were calculated for all protocols and compared to the reference protocol with 26 time frames.

**Results:**

MBF measurements differed (*P* ≤ 0.003) in six (43%) protocols in comparison to the reference protocol, with mean absolute relative differences up to 16% (range 5%-31%). Statistically significant differences were most frequently found for protocols with tissue phase time frames < 90 seconds. MFR did not differ (*P* ≥ 0.11) for any of the protocols.

**Conclusions:**

Various temporal sampling protocols result in different MBF values using Rb-82 PET. MFR measurements were more robust to different temporal sampling protocols.

**Supplementary Information:**

The online version contains supplementary material available at 10.1007/s12350-021-02555-4.

## Introduction

Quantification of myocardial blood flow (MBF) and myocardial flow reserve (MFR) using Rubidium-82 (Rb-82) PET is increasingly used in daily clinical practice. It provides valuable prognostic information in addition to the visual evaluation of myocardial perfusion imaging (MPI) PET data in the detection and evaluation of coronary artery disease (CAD).^1^–^5^ The increasing use of MBF and MFR quantification among multiple hospitals performing Rb-82 PET MPI and the lack of consensus in literature and guidelines on reconstruction protocols has led to a wide variety of temporal sampling protocols that could limit accuracy and data comparison between centers.^6^,^7^

A temporal sampling protocol is used to reconstruct dynamic images. These dynamic images are then used to determine the tracer activity concentration in the blood pool (left ventricle (LV)) and myocardial tissue over time in order to quantify MBF and MFR.^3^ It is important that these measurements are accurate as the resulting time–activity curves (TACs) are used as input for compartmental analysis to calculate the MBF.^3^,^8^,^9^ Both the length and the number of time frames in the temporal sampling protocol may influence the measured TACs and may therefore alter MBF and MFR measurements.^10^ In order to interchange and interpret MBF and MFR values across different centers, it is important to know the effect of temporal sampling on absolute MBF and MFR measurements. Therefore, our aim was to assess the effect of various clinically used temporal sampling protocols on MBF and MFR quantification.

## Materials and Methods

### Temporal Sampling Protocol Selection

A literature search was performed using the Scopus database to find articles available in September 2020. The search strategy to identify all possible temporal sampling protocols used in clinical practice involved the use of the following terms in the title, keywords or abstract: “Rubidium” or “Rb,” and “myocardial blood flow” or “MBF” or “flow,” and “quantification” or “sampling” or “dynamic” or “time frame” or “frame time,” and not “dog” or “canine” or “rabbit,” or “animal.” The full texts of all the articles that were found were screened for temporal sampling protocols used for Rb-82 PET MPI. Exclusion criteria were study populations consisting of animals and phantom or simulation studies. Furthermore, Lee et al. suggest not to use time frames < 5s during the first-pass (blood pool) phase, as these may contain inadequate count statistics.^6^,^10^ Therefore, protocols using time frames < 5s during the first-pass phase were excluded. Protocols using time frames > 10 seconds in the first-pass phase were also excluded as these are likely to result in under-sampling of the left ventricle TAC.^3^

### Study Design

We retrospectively included 20 patients referred for MPI using Rb-82 PET/CT (Vereos, Philips Healthcare) who underwent dynamic rest and regadenoson-induced stress imaging. These 20 patients comprised 10 patients with a scan interpreted as normal by a nuclear medicine physician and 10 in whom the Rb-82 PET scan was interpreted as abnormal (ischemic or irreversible defect). In this way, we ensured the applicability not only in patient scans interpreted as normal but also in patient scans with less perfusion. Approval by the medical ethics committee was not required according to Dutch law as this study was performed retrospectively. Nevertheless, all patients provided written informed consent for the use of their data for research purposes.

### Patient Preparation and Data Acquisition

All subjects were asked to abstain from caffeine-containing substances for 24 hours and to discontinue dipyridamole-containing medication for 48 hours before imaging. Preceding to MPI, a low-dose CT scan was acquired using 1.5 seconds rotation time, a pitch of 0.83, a collimation of 64 × 0.625 mm, a tube voltage of 120 kV, and a tube current of 22 mA. Next, 740 MBq Rb-82 was administered intravenously with a flow rate of 50 mL/min using a Strontium-82/Rb-82 generator (CardioGen-82, Bracco Diagnostics Inc.). Ten minutes after the first elution, we induced pharmacological stress by administrating 400 µg (5 mL) of regadenoson over 10 seconds. After a 5 mL saline flush (NaCl 0.9%), we administered a second dose of 740 MBq Rb-82. We acquired seven-minute PET list-mode acquisitions on the PET system after both Rb-82 administrations. CT-based attenuation correction was applied after registration of CT and PET data.

### Data Processing

CT data were reconstructed using an iterative reconstruction method (iDose level 4) and a slice thickness of 3 mm. PET images were reconstructed with 3D ordered subset expectation maximization (OSEM) using 2 iterations and 15 subsets and a 3D Gaussian post-smoothing filter of 6 mm. Corrections were performed for decay, attenuation, scatter and random coincidences, and dead time effects. The reconstructed dynamic images were post-processed by the same experienced operator using Corridor4DM software (v2017).

**Figure 1:**
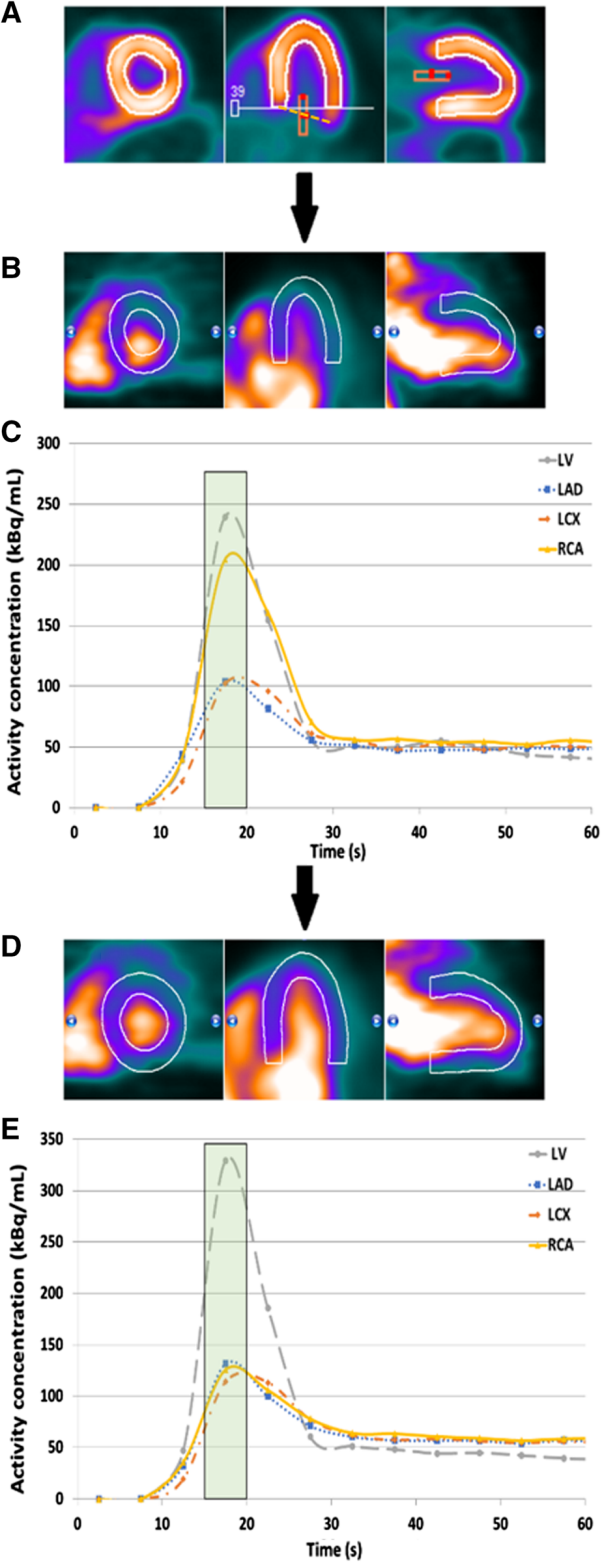
Overview of the three main steps to detect and correct for myocardial movement using Corridor4DM, adapted from Koenders et al. ^11^. The activity concentration in the left ventricle (LV) was measured by the red rectangular region of interest (ROI) which was placed at the center of the imaginary line (yellow dashed line) between the septum and lateral wall, the mitral valve (A). The myocardium contour (white lines in **A**, **B** and **D**) was automatically drawn by assigning the most basal part of the septum which still contains activity. If needed, we corrected for myocardial movement (**B**, **C**) by manually realigning the myocardium contour with the activity in each individual time frame (**D**, **E**)

Myocardium contours were automatically detected in both rest and stress scans based on the static images which were reconstructed from the data acquired between 2:30 and 7:00 minutes (tissue phase). Furthermore, a region of interest (ROI) was automatically placed in the images. If needed, this ROI was manually replaced to the location of the mitral valve to estimate the activity in the blood pool as shown in Figure 1^11^. This was done by assigning an imaginary line between the septal and lateral wall which has to run through the center of the ROI as shown in Figure 1. We manually checked and corrected the dynamic images for the presence or myocardial creep.^12^ The activity concentrations in the myocardium contour and ROI were measured in the reconstructed time frames of the different temporal sampling protocols to calculate the TACs for the LV, the whole myocardium (global), and the three vascular territories: left anterior descending (LAD), left circumflex (LCX), and right coronary artery (RCA). The one-tissue compartment (1-TCM) model of Lortie et al. based on an ROI methodology was used to calculate the absolute MBF from the TACs using Corridor4DM.^13^ Furthermore, the MFR, defined as the stress MBF divided by the rest MBF, was automatically calculated as well.

The protocol stated to be most optimal by Lee et al. uses 26 frames (24 × 5 seconds, 2 × 120 seconds) and was used as reference.^10^ MBF values were excluded in case of an unreliable TAC when using the reference protocol. An unreliable TAC was defined as a TAC without a clear LV peak during the first-pass phase when activity reaches the LV, or a lack of steady state during the tissue phase when the activity is only present in the myocardium, as explained by Koenders et al.^12^ We post-processed the reference protocol (26A) a second time (26A*) to ascertain the reproducibility of post-processing the data. Absolute relative differences in rest MBF, stress MBF, and MFR measurements as compared to the values obtained using the reference protocol were calculated and classified into two categories: ≤ 10% and > 10%.

### Statistical Analysis

Patient-specific parameters and characteristics were determined as percentage or mean ± standard deviation (SD). For each patient, we calculated rest MBF, stress MBF, and MFR for the reference protocol as well as for the 14 selected protocols. We compared these three measurements for each of the 14 protocols to the reference protocol using the Wilcoxon signed rank test using SPSS Statistics version 24.0 (IBM Corporation). Following a Bonferroni correction for the 14 different comparisons, the level of statistical significance was set to 0.05/14 = 0.004 for all statistical analyses.

## Results

**Figure 2:**
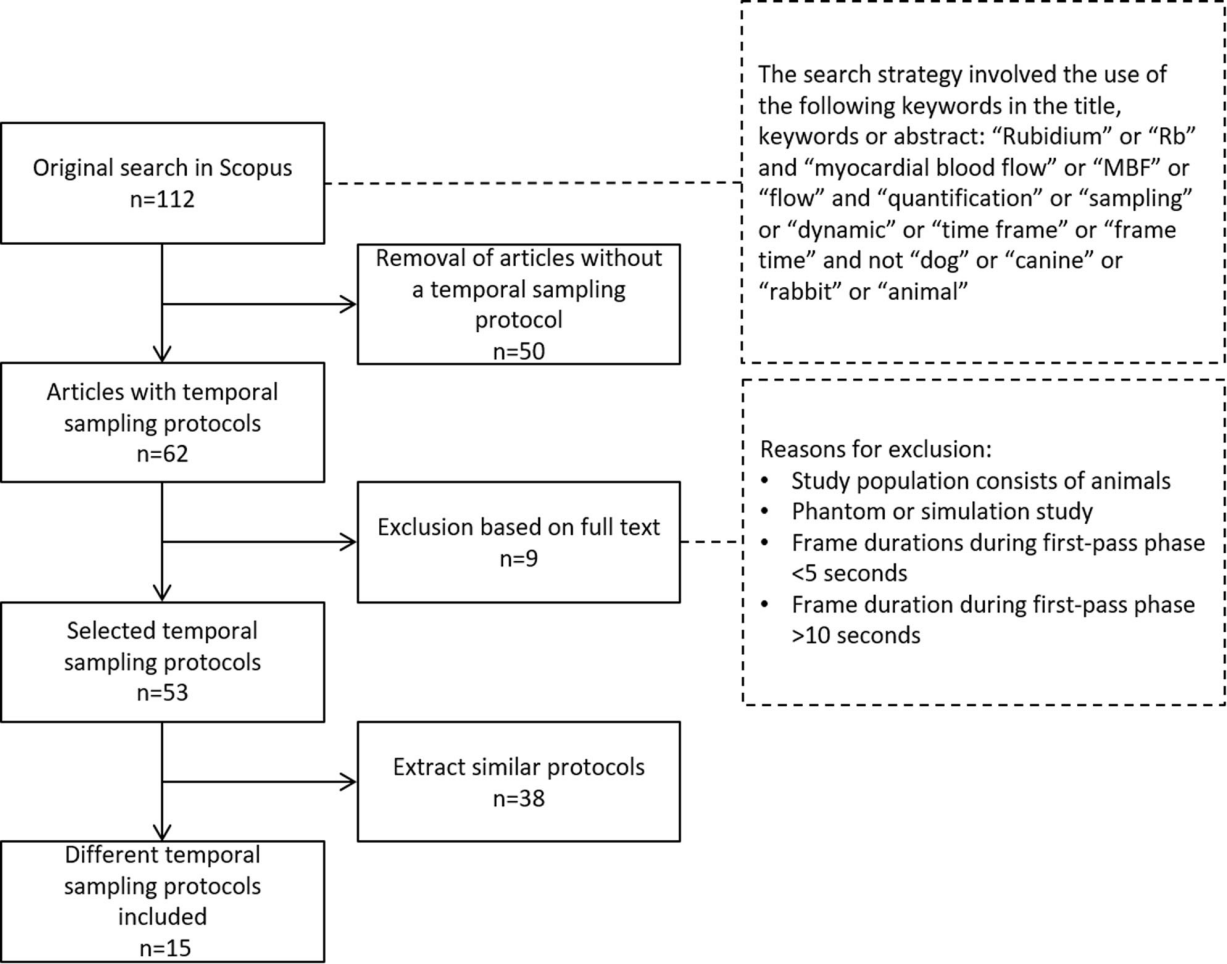
Flow chart of temporal sampling protocol selection. The full texts of all the articles that were found were screened for temporal sampling protocols used for Rb-82 PET MPI

**Figure 3:**
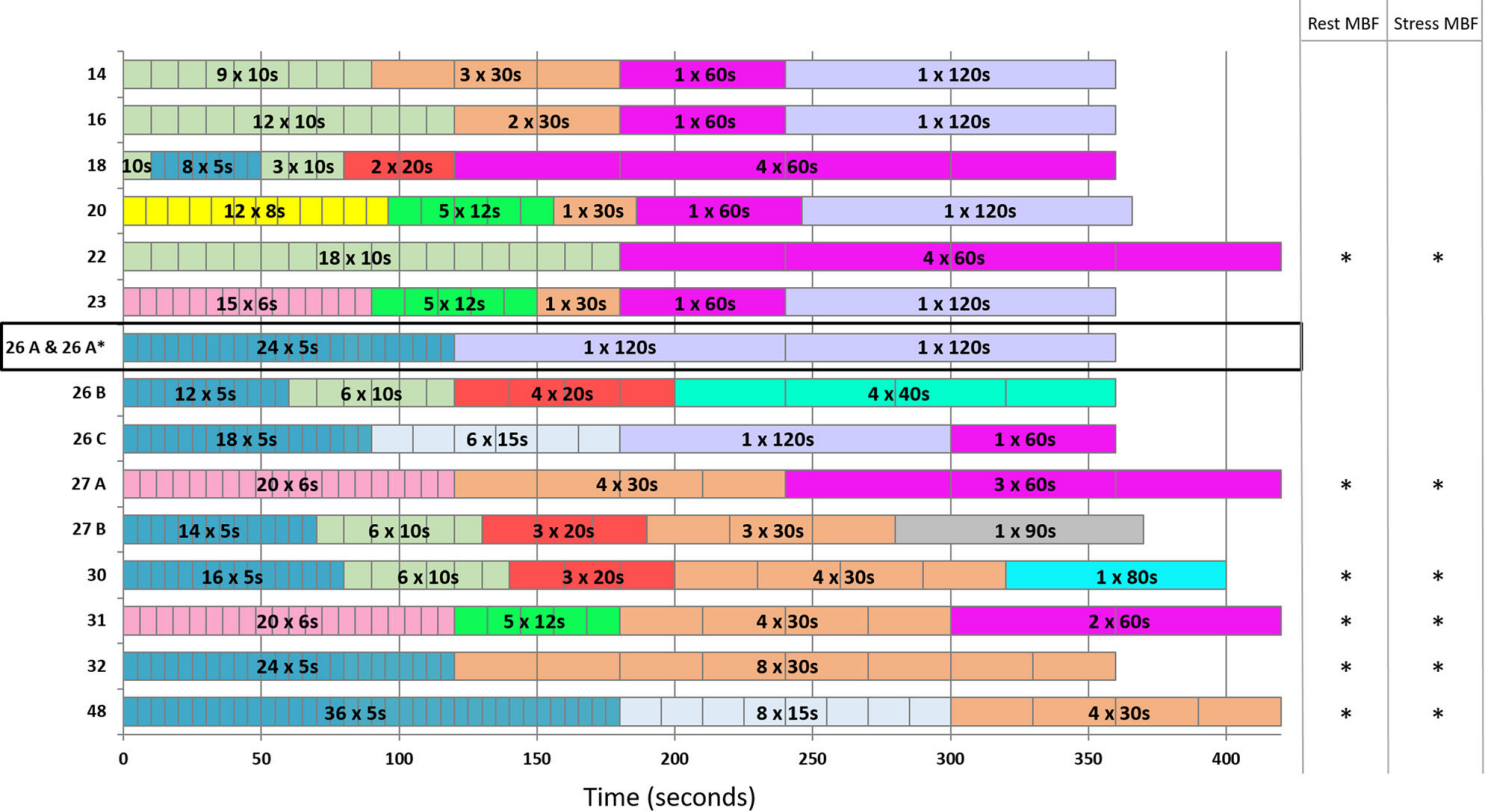
Temporal sampling protocols that were tested and compared to the reference protocol (26A) (in the black box) that was post-processed twice (26A*). The number of frames of each protocol is shown on the y-axis and the time in seconds on the x-axis. Every frame has a color representing the duration of that frame, for example, yellow represents 8-second time frames and pink represents 60-second time frames. Significant differences in global rest or stress MBF (mL/min/g) values compared to the reference protocol are indicated on the second y-axis (*P< 0.004). No significant differences in global MFR were found

We screened 112 articles finding 62 potentially relevant articles containing temporal sampling protocols, as shown in Figure 2. Upon additional review, this resulted in 15 different temporal sampling protocols that were applied to patient data, including the reference protocol referred by 26A, as shown in Table 1 and Figure 3. The baseline characteristics of the included patients are summarized in Table 2.

**Table 1:** Overview of the 14 tested temporal sampling protocols and the reference protocol (26A) that was post-processed twice (26A*)

Number of frames	Frame lengths				
14	9 × 10 seconds	3 × 30 seconds	1 × 60 seconds	1 × 120 seconds	
16	12 × 10 seconds	2 × 30 seconds	1 × 60 seconds	1 × 120 seconds	
18	1 × 10 seconds	8 × 5 seconds	3 × 10 seconds	2 × 20 seconds	4 × 60 seconds
20	12 × 8 seconds	5 × 12 seconds	1 × 30 seconds	1 × 60 seconds	1 × 120 seconds
22	18 × 10 seconds	4 × 60 seconds			
23	15 × 6 seconds	5 × 12 seconds	1 × 30 seconds	1 × 60 seconds	1 × 120 seconds
26A & 26A*	24 × 5 seconds	2 × 120 seconds			
26B	12 × 5 seconds	6 × 10 seconds	4 × 20 seconds	4 × 40 seconds	
26C	18 × 5 seconds	6 × 15 seconds	1 × 120 seconds	1 × 60 seconds	
27A	20 × 6 seconds	4 × 30 seconds	3 × 60 seconds		
27B	14 × 5 seconds	6 × 10 seconds	3 × 20 seconds	3 × 30 seconds	1 × 90 seconds
30	16 × 5 seconds	6 × 10 seconds	3 × 20 seconds	4 × 30 seconds	1 × 80 seconds
31	20 × 6 seconds	5 × 12 seconds	4 × 30 seconds	2 × 60 seconds	
32	24 × 5 seconds	8 × 30 seconds			
48	36 × 5 seconds	8 × 15 seconds	4 × 30 seconds

**Table 2:** Baseline characteristics of all included patients presented as mean ± SD or percentage

Characteristic	All patients (n=20)
Age (years)	67 ± 9
Male gender (%)	80
Weight (kg)	87 ± 15
Length (cm)	177 ± 8
BMI (kg⋅m^2^)	27.7 ± 4.3
Current smoker (%)	5
Hypertension (%)	45
Dyslipidaemia (%)	40
Diabetes (%)	30
Family history (%)	55

**Figure 4:**
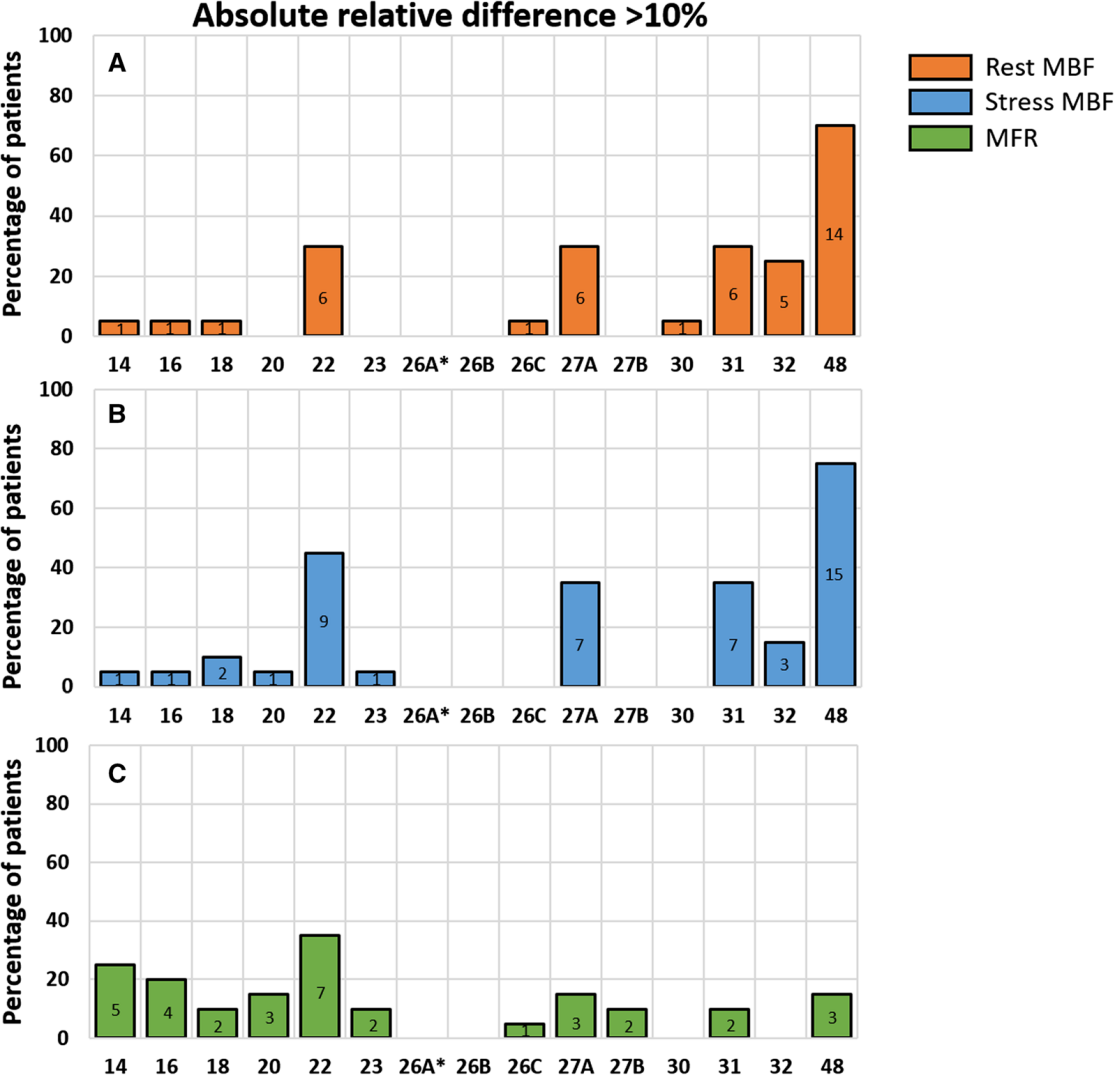
Barplot showing the percentage of patients with absolute relative differences >10% in **A** global rest MBF (mL/min/g), **B** global stress MBF (mL/min/g), and **C** global MFR for all tested protocols as compared to the reference protocol that was post-processed twice (26A*). The absolute number of patients is shown within the bars

**Figure 5:**
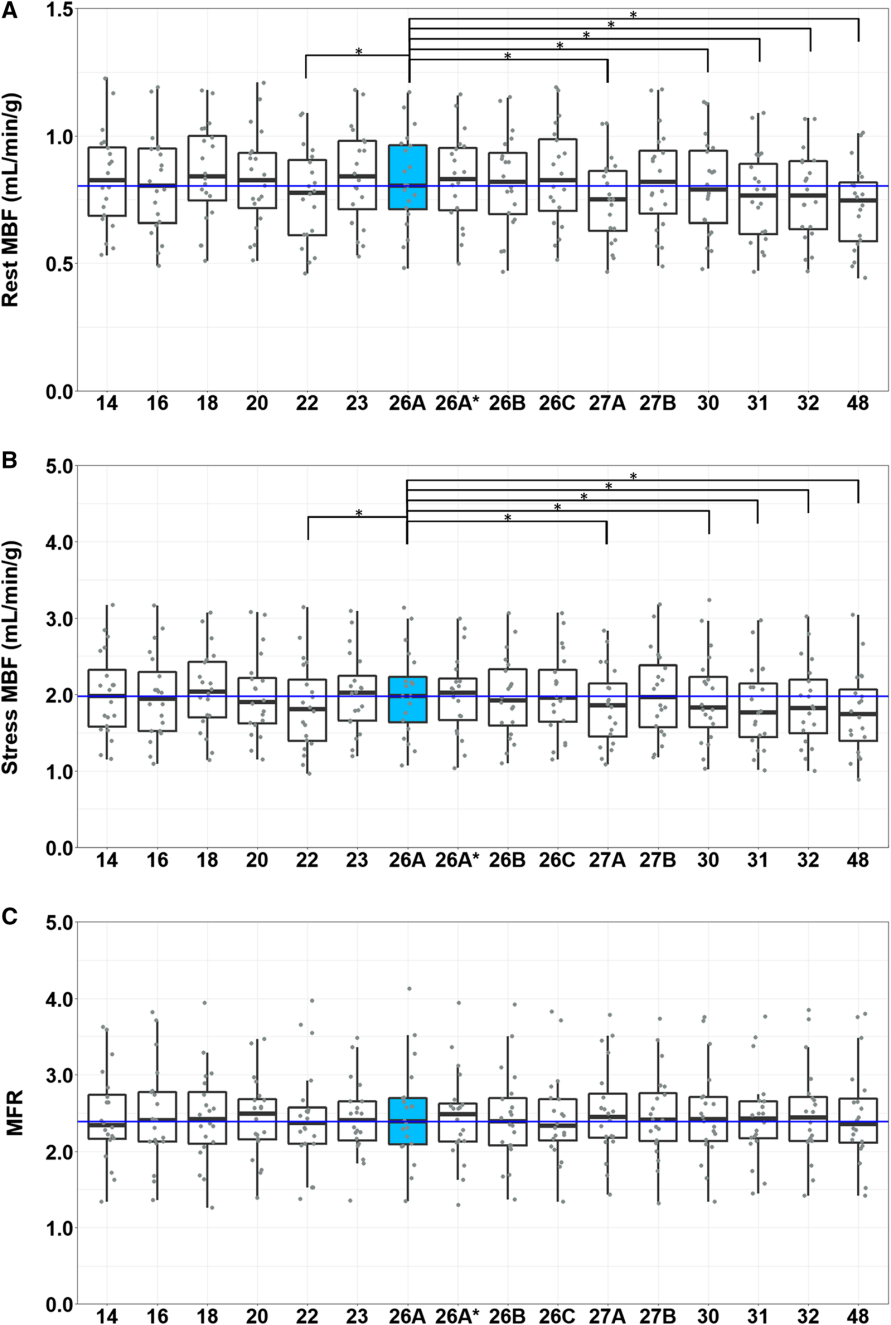
Boxplots of the 14 tested temporal sampling protocols and the boxplot showing the reproducibility (26A*) with the MBF and MFR values of each patient (gray dots) and the median value (dark blue line) representing the reference protocol (26A, blue) for global rest (**A**) and stress (**B**) MBF and MFR (**C**). *P < 0.004

We found a good reproducibility for the reference protocol as the mean absolute relative differences were ≤ 4.1% as shown in Figure 4. Neither the MBF nor the MFR measurements differed significantly after Bonferroni correction (*P* > 0.01), as shown in Figure 5.

Six out of the 14 (43%) temporal sampling protocols resulted in different global rest and stress MBF (*P* ≤ 0.003) as compared to the reference protocol. The corresponding protocols were those with 22, 27 (A), 30, 31, 32, and 48 frames as shown in Table 3 and Figure 5. Significant differences in MBF were found for these six protocols which all use time frames < 90 seconds in the tissue phase instead of 120 seconds as used in the reference protocol. Compared to the reference, none of the tested protocols showed a difference (*P* ≥ 0.15) in global MFR measurements. Median values with interquartile ranges of the MBF and MFR measurements for all 20 patients obtained using the different temporal sampling protocols and the mean absolute relative differences to the reference protocol with ranges are given in Table 3.

**Table 3:** Global flow values and mean absolute relative differences for all 14 protocols in comparison to the reference protocol that was post-processed twice (26A and 26A*)

	Global					
	Flow values			Mean absolute relative difference		
	Stress MBF (mL/min/g)	Rest MBF (mL/min/g)	MFR	Stress MBF (%)	Rest MBF (%)	MFR (%)
14	1.98 [1.55–2.49]	0.83 [0.68–0.97]	2.34 [2.15–2.80]	4.9 (1–13)	3.7 (0–11)	6.8 (0–16)
16	1.95 [1.52–2.41]	0.81 [0.65–0.95]	2.40 [2.13–2.78]	4.2 (1–14)	4.1 (0–11)	6.5 (1–17)
18	2.04 [1.67–2.44]	0.84 [0.72–1.02]	2.42 [2.07–2.85]	4.0 (0–15)	5.1 (0–15)	6.2 (1–15)
20	1.90 [1.61–2.26]	0.83 [0.69–0.94]	2.49 [2.14–2.71]	3.9 (0–12)	3.6 (0–10)	4.8 (0–17)
22	1.81 [1.37–2.33]*	0.78 [0.61–0.92]*	2.37 [2.10–2.63]	9.7 (0–20)	8.0 (1–19)	7.2 (0–18)
23	2.03 [1.65–2.37]	0.84 [0.68–0.98]	2.40 [2.11–2.65]	3.3 (0–11)	3.6 (0–10)	5.5 (1–19)
26A	1.98 [1.58–2.36]	0.81 [0.70–0.97]	2.39 [2.07–2.70]	Ref	Ref	Ref
26A*	2.02 [1.57–2.29]	0.83 [0.70–0.96]	2.48 [2.11–2.65]	3.3 (1–5)	2.1 (0–5)	3.0 (0–9)
26B	1.93 [1.52–2.37]	0.82 [0.68–0.96]	2.39 [2.08–2.73]	3.2 (0–8)	2.8 (0–7)	3.3 (0-10)
26C	1.96 [1.62–2.40]	0.83 [0.68–1.00]	2.33 [2.09–2.69]	3.1 (0–7)	2.7 (0–13)	3.7 (0–13)
27A	1.86 [1.39–2.26]*	0.75 [0.6–0.87]*	2.45 [2.1–2.84]	8.2 (1–18)	9.3 (1–34)	7.0 (0–36)
27B	1.97 [1.54–2.45]	0.82 [0.67–0.97]	2.41 [2.1–2.81]	3.9 (0–10)	3.0 (0–6)	4.7 (0–14)
30	1.83 [1.48–2.29]*	0.79 [0.63–0.95]*	2.42 [2.1–2.71]	4.2 (0–9)	3.8 (0–12)	3.3 (0-9)
31	1.77 [1.42–2.26]*	0.77 [0.61–0.91]*	2.42 [2.1–2.65]	9.0 (2–17)	8.0 (2–16)	5.7 (0-15)
32	1.83 [1.43–2.25]*	0.77 [0.63–0.77]*	2.44 [2.13-2.73]	7.2 (1–14)	7.6 (1–14)	4.1 (0-10)
48	1.74 [1.29–2.17]*	0.75 [0.58–0.83]*	2.35 [2.08-2.76]	13.2 (3–23)	12.7 (5–20)	5.9 (0-16)

Data are presented as median [interquartile range] and mean absolute relative difference (minimum–maximum).

Significant differences in MBF and MFR measurements as compared to the reference protocol are indicated with * (*P*<0.004).

*MBF*, myocardial blood flow; *MFR*, myocardial flow reserve

Absolute relative differences in both rest and stress MBF were ≤ 10% in all patients for protocols using 26 (B) and 27 (B) frames with mean absolute relative differences up to 4% as shown in Table 3 and Figure 4. In addition, protocols using 20, 23, 26 (C), and 30 frames showed absolute relative differences of ≤ 10% for just rest or stress MBF.

On a regional level, both rest and stress MBF differed (*P* ≤ 0.002) in all regional territories (LAD, LCX, and RCA) for the protocols using 22, 27 (A), 31, 32, and 48 frames. Median values with interquartile ranges of regional MBF and MFR values obtained using the different temporal sampling protocols and the mean absolute relative differences to the reference protocol with ranges are given in the Appendix. Results on a regional level were in agreement with the results found for global rest and stress MBF. Compared to the reference, none of the tested protocols showed a significant difference (*P* ≥ 0.11) in regional MFR measurements.

## Discussion

In this study, we selected temporal sampling protocols used in Rb-82 PET MPI from the literature and assessed the effect on absolute blood flow measurements. We showed that the use of various temporal sampling protocols can result in different rest and stress MBF, both on a regional and global level. We found mean absolute relative differences up to 13% for global MBF and up to 16% for regional MBF in comparison to the reference protocol. No significant differences were found for global or regional MFR.

Several studies have reported the importance of accurate temporal sampling of the first-pass phase for MBF quantification.^3^,^8^–^10^ The temporal sampling protocol that we used as reference was stated by Lee et al. to optimally sample the blood pool TAC.^10^ This protocol uses 24 5-second frames for the first-pass and intermediate (activity in both LV and myocardium) phase. Furthermore, the protocol uses 2 frames of 120 seconds for the tissue phase (activity mainly present in the myocardium) as it was shown that such long frame durations hardly affect MBF measurements.^10^ In our study, differences in MBF were most frequently found for the protocols with time frames less than 90 seconds in the tissue phase instead of 120 seconds as used in the reference protocol. More specifically, is seems that small variations in the input function alter MBF measurements, presumably due to insufficient count statistics during the tissue phase. Yet the protocol with 18 frames was the only protocol that uses time frames less than 90 seconds for which we did not find a significant difference in MBF. As we found a good reproducibility for the reference protocol, it is unlikely that the manual relocation of the ROI or the myocardial contours caused the differences in MBF measurements. Possibly, deviating MBFs might partly be explained by time frames which are too small or too large in the intermediate phase in combination with shorter time frames in the tissue phase. The effect of time framing during the first-pass phase was expected to be limited as we only used frames of 5-10 seconds in this phase.^10^

Compared to the reference, none of the tested protocols showed a significant difference in global or regional MFR measurements. As the MFR is the ratio between stress and rest MBF, it seems to correct for systemic biases of rest and stress MBF introduced by several temporal sampling protocols (Figure 5). However, as MFR is defined as the ratio between stress and rest MBF, error propagation might cause the variance of MFR measurements to exceed the variance of MBF measurements.^14^ This likely explains that for some protocols (14, 16, and 20), we observed more patients with an absolute relative difference > 10% for MFR than for rest or stress MBF (Figure 4). Furthermore, conflicting studies exist regarding the preference for stress MBF or MFR for risk stratification of patients with suspected CAD. Several studies found that stress MBF is superior to MFR,^15^–^17^ while others found that the MFR is superior to stress MBF for risk stratification.^1^,^3^–^5^,^18^ Murthy et al.^19^ and Tahari et al.^20^ reported that MFR was more consistent when different software is used and when different methods are used to determine the input function. Moreover, MFR was shown to be more robust in case of different advanced reconstruction settings as compared to MBF values.^21^ In our study, MFR measurements were clearly less dependent on the temporal sampling protocol as compared to MBF measurements, which supports MFR as the preferred parameter.

There are several limitations to this study that should be recognized. First, it was not possible to evaluate the effect of different temporal sampling protocols on the diagnostic accuracy due to the lack of a reference standard and the relatively small sample size. However, we did use an optimized temporal sampling protocol suggested by Lee et al. as a reference^10^ and performed a pair-wise comparison, limiting the need for a large sample size.

Secondly, we used a relatively low Rb-82 activity (740 MBq) as compared to the generally recommended activity of 1110 MBq.^3^ This relatively low amount of activity is sufficient for MBF quantification using the Vereos PET scanner which contains sensitive silicon photomultipliers with digital readout.^22^,^23^ Higher activities than 740 MBq will presumably result in better count statistics during the tissue phase and could therefore result in a better image quality. However, higher activities may hamper blood flow quantification when using PET scanners with photomultiplier tubes that have a low count-rate performance.^22^,^24^ If the activities administered exceed the dynamic range of the PET scanner, it will lead to an underestimation of the count-rate during the first-pass phase which will influence the TAC^10^,^22^,^24^ and therefore alter flow measurements. Nevertheless, if we had used a higher activity in all our patients, the count statistics during the tissue phase would be better which would possibly result in less MBF variation when using protocols with shorter time frames in the tissue phase as compared to the reference protocol. However, as previous studies showed that there is no added value in using shorter time frames during the tissue phase, we do not recommend these protocols.^10^

Finally, we tested temporal sampling protocols only using the 1-TCM of Lortie et al.^13^ as this model is most commonly used for Rb-82. Therefore, we did not include other 1-TCMs with different extraction functions^25^ or other compartmental models such as the two-compartment model^26^ and the retention model.^27^,^28^ The retention model is a simpler model as compared to the 1-TCM^27^,^29^ as it “does not use TACs, but instead integrates arterial input and myocardial uptake over the first 2 and the following 5 minutes, respectively, after tracer injection.”^30^ It was already shown that the use of the retention model resulted in differences in stress MBF when compared to the 1-TCM in combination with the ROI methodology and therefore the two models cannot be used interchangeably.^19^,^20^

## New Knowledge Gained

This manuscript provides new insights and has several clinical consequences. First, one should be cautious in using different temporal sampling protocols in PET imaging as we found significant differences for rest and stress MBF measurements in the myocardium as a whole but also on a regional level. It seems that MFR is less dependent on temporal sampling (this study) and also on other technical variations.^19^–^21^ Therefore, MFR seems to be a more suitable parameter to be used between centers and for multicentre trials. To use rest and stress MBF among multiple sites in the detection of CAD and in multicentre trials, harmonization of all technical aspects such as temporal sampling is necessary.

## Conclusions

Various temporal sampling protocols for MBF and MFR quantification using Rb-82 PET result in different MBF values. MFR measurements were more robust to different temporal sampling protocols. Hence, we recommend using MFR instead of MBF measurements, especially when employed at different centers and in multicenter trials.

### Supplementary Information

Below is the link to the electronic supplementary material.

Supplementary file1 (PPTX 3366 kb)

## Appendix

See Tables

**Table 4:** Flow values in the LAD territory and mean absolute relative differences for all 14 protocols in comparison to the reference protocol that was post-processed twice (26A and 26A*).

	LAD					
	Flow values			Mean absolute relative difference		
	Stress MBF (mL/min/g)	Rest MBF (mL/min/g)	MFR	Stress MBF (%)	Rest MBF(%)	MFR (%)
14	1.96 [1.53–2.39]	0.83 [0.63-0.93]	2.28 [2.05-2.90]	5.0 (1-11)	5.8 (0-18)	10.0 (1-23)
16	1.95 [1.49-2.41]	0.80 [0.62–0.92]	2.41 [2.10–2.87]	4.5 (0–10)	6.2 (1–27)	9.8 (0–46)
18	1.97 [1.56–2.38]	0.84 [0.70–0.97]	2.41 [2.06–2.89]	3.9 (0–15)	4.9 (1–14)	6.6 (0–15)
20	1.94 [1.51–2.32]	0.80 [0.68–0.95]	2.43 [2.16–2.80]	4.4 (1–14)	5.0 (0–12)	6.7 (0–19)
22	1.88 [1.38–2.31]*	0.76 [0.57–0.89]*	2.44 [2.12–2.83]	7.9 (0–16)	9.1 (0–35)	11.4 (0–34)
23	2.00 [1.52–2.34]	0.82 [0.68–0.99]	2.37 [2.09–2.75]	3.8 (1–13)	4.2 (0–10)	6.7 (0–21)
26A	2.00 [1.48–2.28]	0.80 [0.72–0.94]	2.41 [2.09–2.67]	Ref	Ref	Ref
26A*	1.96 [1.50–2.22]	0.82 [0.71–0.94]	2.37 [2.02–2.65]	3.3 (0–7)	2.2 (0–6)	4.1 (0–10)
26B	1.94 [1.48–2.29]	0.82 [0.67–0.94]	2.43 [2.01–2.75]	3.1 (0–9)	4.1 (0–11)	4.8 (0–13)
26C	1.95 [1.48–2.36]	0.84 [0.69–0.97]	2.35 [1.99–2.76]	2.9 (0–10)	4.2 (0–16)	4.5 (0–17)
27A	1.84 [1.41–2.26]*	0.75 [0.59–0.86]*	2.47 [2.07–2.73]	7.5 (1–19)	8.8 (0–20)	6.5 (0–23)
27B	1.94 [1.48–2.39]	0.83 [0.63–0.96]	2.49 [2.09–2.83]	3.8 (0–10)	4.5 (0–16)	6.3 (0–15)
30	1.85 [1.40–2.29]	0.81 [0.60–0.92]*	2.47 [2.14–2.70]	4.1 (1–10)	5.3 (0–20)	6.2 (1–25)
31	1.81 [1.39–2.21]*	0.77 [0.58–0.84]*	2.39 [2.14–2.69]	7.5 (1–17)	9.0 (2–23)	7.2 (1–20)
32	1.87 [1.38–2.25]*	0.76 [0.60–0.85]*	2.41 [2.12–2.76]	6.9 (1–15)	9.1 (2–24)	6.4 (1–22)
48	1.78 [1.33–2.12]*	0.74 [0.56–0.80]*	2.36 [2.08–2.78]	11.7 (3–23)	13.1 (1–28)	9.0 (2–23)

Data are presented as median [interquartile range] and mean absolute relative difference (minimum–maximum).

Significant differences in MBF and MFR measurements as compared to the reference protocol are indicated with * (*P*<0.004).

*MBF*, myocardial blood flow; *MFR*, myocardial flow reserve

4,

**Table 5:** Flow values in the LCX territory and mean absolute relative differences for all 14 protocols in comparison to the reference protocol that was post–processed twice (26A and 26A*).

	LCX					
	Flow values			Mean absolute relative difference		
	Stress MBF (mL/min/g)	Rest MBF (mL/min/g)	MFR	Stress MBF (%)	Rest MBF (%)	MFR (%)
14	1.87 [1.51–2.26]	0.85 [0.69–1.00]	2.29 [1.86–2.85]	4.2 (0–13)	4.7 (0–14)	6.9 (1–19)
16	1.82 [1.46–2.21]	0.82 [0.67–1.00]	2.31 [1.92–2.82]	4.5 (1–14)	5.3 (0–16)	6.4 (0–20)
18	1.86 [1.56–2.20]	0.88 [0.74–1.00]	2.19 [1.98–2.85]	4.2 (0–14)	4.9 (0–15)	7.4 (1–15)
20	1.79 [1.49–2.17]	0.86 [0.69–1.01]	2.33 [1.81–2.78]	3.6 (0–10)	4.2 (0–13)	5.2 (0–26)
22	1.68 [1.29–2.10]*	0.78 [0.61–0.94]*	2.19 [1.94–2.79]	11.3 (1–22)	10.2 (0–28)	8.3 (1–18)
23	1.92 [1.52–2.19]	0.87 [0.71–1.01]	2.17 [1.93–2.77]	3.3 (0–7)	4.1 (0–13)	5.4 (0–16)
26A	1.86 [1.45–2.21]	0.85 [0.72–0.98]	2.12 [1.82–2.94]	Ref	Ref	Ref
26A*	1.87 [1.52–2.17]	0.85 [0.73–0.96]	2.16 [1.89–2.93]	3.3 (0–7)	1.9 (0–8)	3.7 (1–8)
26B	1.79 [1.40–2.19]*	0.85 [0.70–0.96]	2.16 [1.82–2.97]	3.6 (0–7)	3.6 (0–11)	3.5 (0–10)
26C	1.85 [1.46–2.28]	0.86 [0.71–1.04]	2.17 [1.93–2.76]	3.6 (0–10)	3.4 (0–13)	5.1 (0–12)
27A	1.73 [1.39–2.06]*	0.79 [0.62–0.94]*	2.25 [2.00–2.85]	8.8 (0–16)	9.6 (1–25)	6.8 (0–22)
27B	1.87 [1.46–2.33]	0.87 [0.70–0.96]	2.16 [1.91–2.94]	4.4 (1–13)	3.7 (0–8)	4.5 (0–15)
30	1.75 [1.38–2.18]*	0.81 [0.65–0.99]*	2.22 [1.89–2.99]	4.6 (0–10)	5.3 (0–16)	4.2 (0–16)
31	1.61 [1.31–2.08]*	0.79 [0.61–0.92]*	2.21 [1.95–2.75]	9.7 (1–19)	9.1 (1–22)	7.7 (2–15)
32	1.69 [1.32–2.15]*	0.79 [0.63–0.93]*	2.26 [1.92–2.96]	7.4 (0–15)	8.7 (2–20)	5.1 (0–18)
48	1.61 [1.22–2.00]*	0.73 [0.58–0.85]*	2.18 [1.93–2.93]	13.9 (1–23)	14.3 (6–26)	6.4 (1–15)

Data are presented as median [interquartile range] and mean absolute relative difference (minimum–maximum).

Significant differences in MBF and MFR measurements as compared to the reference protocol are indicated with * (*P*<0.004).

*MBF*, myocardial blood flow; *MFR*, myocardial flow reserve

5, and

**Table 6:** Flow values in the RCA territory and mean absolute relative differences for all 14 protocols in comparison to the reference protocol that was post–processed twice (26A and 26A*).

	RCA					
	Flow values			Mean absolute relative difference		
	Stress MBF (mL/min/g)	Rest MBF (mL/min/g)	MFR	Stress MBF (%)	Rest MBF (%)	MFR (%)
14	2.14 [1.91–2.14]	0.91 [0.76–1.07]	2.62 [2.09–2.87]	6.9 (0–16)	5.4 (0–19)	7.8 (0–15)
16	2.12 [1.83–2.70]	0.94 [0.71–1.06]	2.64 [2.09–2.87]	6.5 (0–18)	5.9 (0–15)	7.7 (1–19)
18	2.33 [1.94–2.91]	0.96 [0.75–1.18]	2.54 [2.09–2.93]	4.5 (0–19)	5.1 (0–18)	6.8 (0–19)
20	2.17 [1.86–2.59]*	0.93 [0.69–1.04]	2.46 [2.30–2.98]	5.7 (0–14)	4.9 (0–16)	5.3 (0–16)
22	1.96 [1.66–2.60]*	0.85 [0.66–0.98]*	2.50 [2.13–2.90]	12.2 (0–27)	10.6 (1–22)	8.6 (0–28)
23	2.22 [1.89–2.72]	0.95 [0.71–1.09]	2.41 [2.15–2.91]	4.4 (0–17)	3.7 (0–10)	5.8 (0–22)
26A	2.38 [1.86–2.72]	0.97 [0.71–1.10]	2.40 [2.15–3.08]	Ref	Ref	Ref
26A*	2.35 [1.94–2.76]	0.97 [0.72–1.08]	2.41 [2.23–3.03]	3.7 (1–11)	3.4 (0–7)	3.2 (0–14)
26B	2.22 [1.87–2.67]*	0.94 [0.71–1.11]	2.43 [2.04–2.99]	5.1 (0–16)	4.1 (0–13)	4.8 (1–16)
26C	2.31 [1.84–2.68]	0.95 [0.69–1.10]	2.34 [2.20–2.84]	3.4 (0–12)	3.3 (0–14)	5.6 (0–23)
27A	2.01 [1.75–2.59]*	0.87 [0.66–1.01]*	2.42 [2.18–2.94]	10.6 (0–22)	9.6 (2–19)	6.3 (0–19)
27B	2.23 [1.83–2.79]	0.94 [0.68–1.10]	2.53 [2.06–2.97]	5.5 (1–14)	4.0 (0–12)	6.9 (0–19)
30	2.19 [1.80–2.57]*	0.90 [0.67–1.03]*	2.51 [2.14–3.06]	6.3 (1–19)	6.9 (0–18)	5.5 (0–15)
31	2.02 [1.73–2.50]*	0.85 [0.64–1.00]*	2.43 [2.22–2.96]	11.4 (3–21)	10.8 (0–22)	6.9 (1–20)
32	2.07 [1.73–2.49]*	0.88 [0.66–1.01]*	2.44 [2.18–2.99]	9.7 (3–21)	9.6 (0–21)	5.7 (0–18)
48	1.95 [1.59–2.40]*	0.80 [0.61–0.93]*	2.40 [2.22–3.05]	16.4 (5–31)	15.9 (7–29)	7.5 (0–24)

Data are presented as median [interquartile range] and mean absolute relative difference (minimum-maximum).

Significant differences in MBF and MFR measurements as compared to the reference protocol are indicated with * (*P* < 0.004).

*MBF*, myocardial blood flow; *MFR*, myocardial flow reserve

6.

## Abbreviations

LAD: Left anterior descending
LCX: Left circumflex
LV: Left ventricle
MBF: Myocardial blood flow
MFR: Myocardial flow reserve
MPI: Myocardial perfusion imaging
PET: Positron emission tomography
Rb-82: Rubidium-82
RCA: Right coronary artery
TAC: Time–activity curve
