# Supplementary material for: Effect of temporal sampling protocols on myocardial blood flow measurements using Rubidium-82 PET
Source: J Nucl Cardiol. 2021 Mar 2;29(4):1729–41. doi: 10.1007/s12350-021-02555-4 (PMC9345838; doi:10.1007/s12350-021-02555-4)
Supplement: Supplementary file 1 — Supplementary file1 (PPTX 3366 kb) [file 12350_2021_2555_MOESM1_ESM.pptx]

## Slide 1
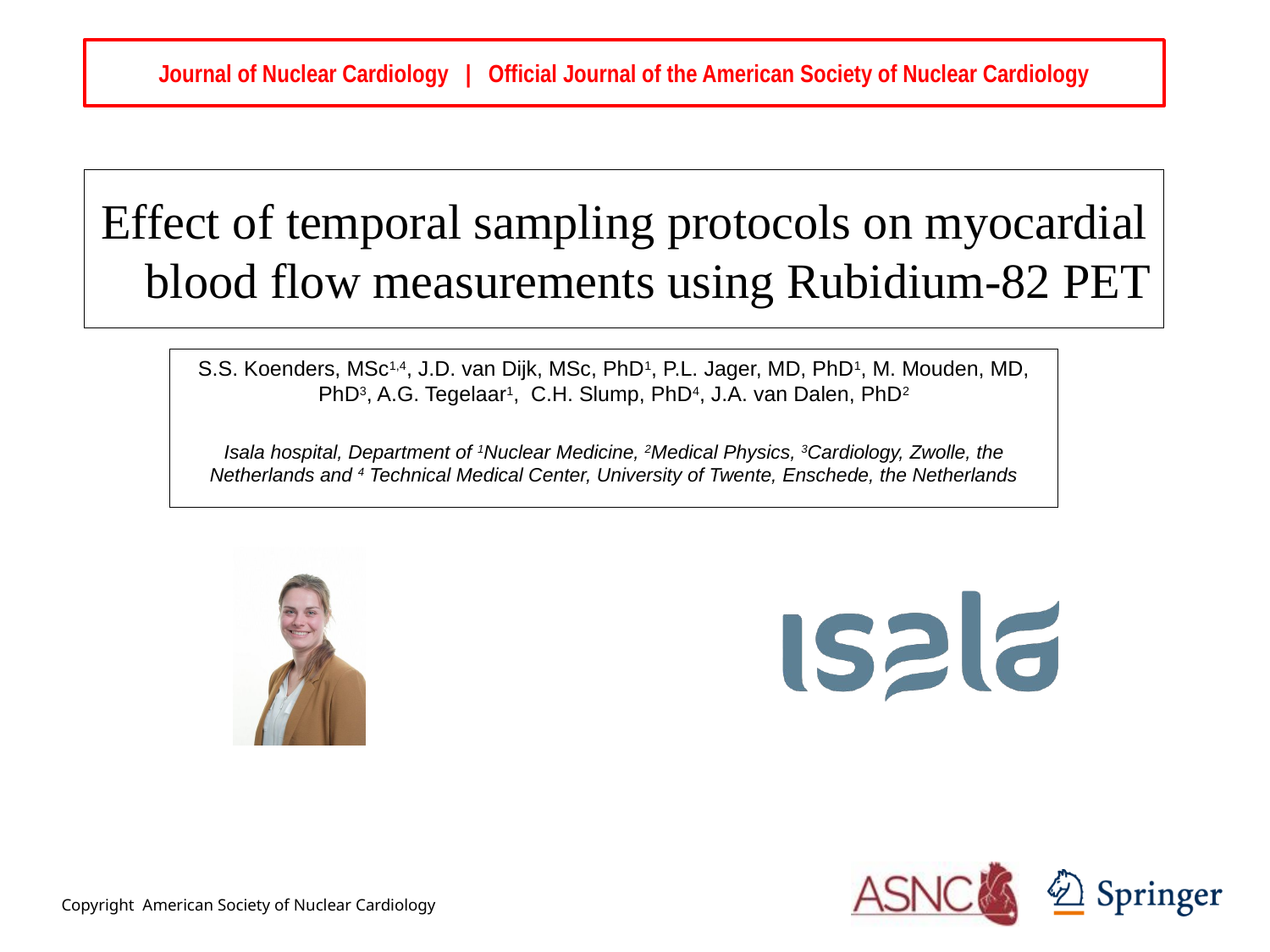

Journal of Nuclear Cardiology | Official Journal of the American Society of Nuclear Cardiology
# Effect of temporal sampling protocols on myocardial blood flow measurements using Rubidium-82 PET
S.S. Koenders, MSc1,4, J.D. van Dijk, MSc, PhD1, P.L. Jager, MD, PhD1, M. Mouden, MD, PhD3, A.G. Tegelaar1, C.H. Slump, PhD4, J.A. van Dalen, PhD2
Isala hospital, Department of 1Nuclear Medicine, 2Medical Physics, 3Cardiology, Zwolle, the Netherlands and 4 Technical Medical Center, University of Twente, Enschede, the Netherlands
Copyright American Society of Nuclear Cardiology

## Slide 2
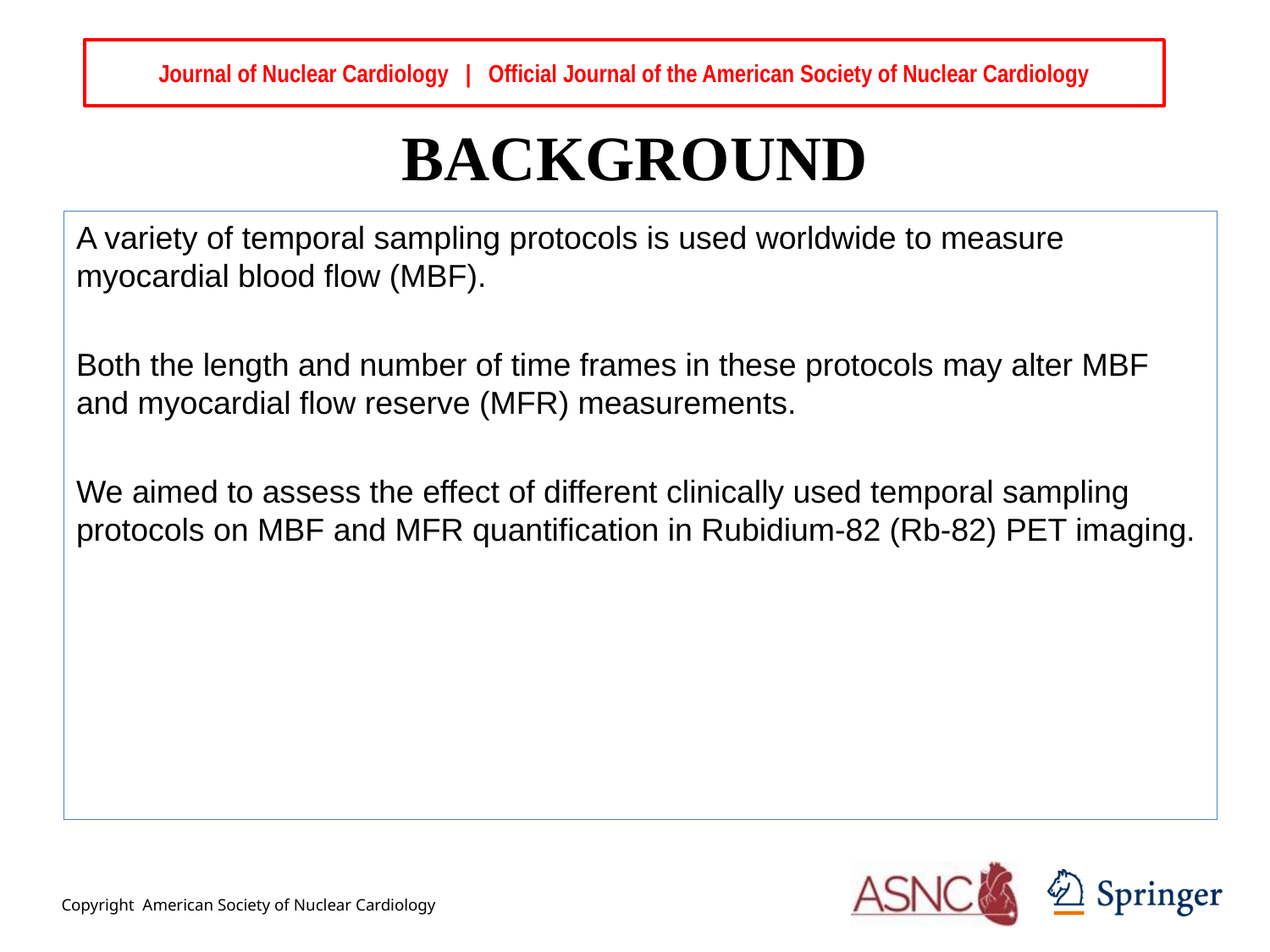

Journal of Nuclear Cardiology | Official Journal of the American Society of Nuclear Cardiology
# BACKGROUND
A variety of temporal sampling protocols is used worldwide to measure myocardial blood flow (MBF).
Both the length and number of time frames in these protocols may alter MBF and myocardial flow reserve (MFR) measurements.
We aimed to assess the effect of different clinically used temporal sampling protocols on MBF and MFR quantification in Rubidium-82 (Rb-82) PET imaging.
Copyright American Society of Nuclear Cardiology

## Slide 3
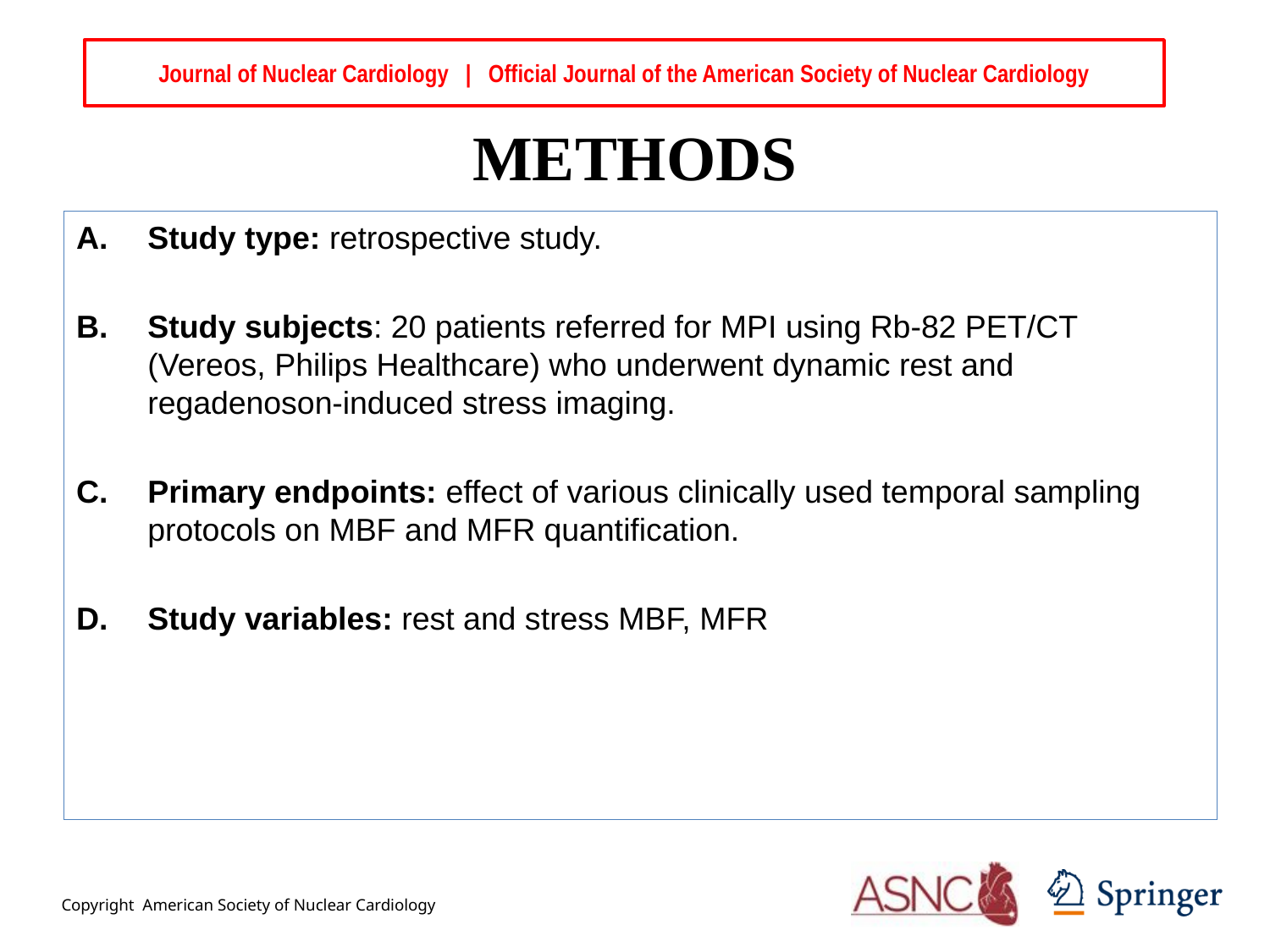

Journal of Nuclear Cardiology | Official Journal of the American Society of Nuclear Cardiology
# METHODS
Study type: retrospective study.
Study subjects: 20 patients referred for MPI using Rb-82 PET/CT (Vereos, Philips Healthcare) who underwent dynamic rest and regadenoson-induced stress imaging.
Primary endpoints: effect of various clinically used temporal sampling protocols on MBF and MFR quantification.
Study variables: rest and stress MBF, MFR
Copyright American Society of Nuclear Cardiology

## Slide 4
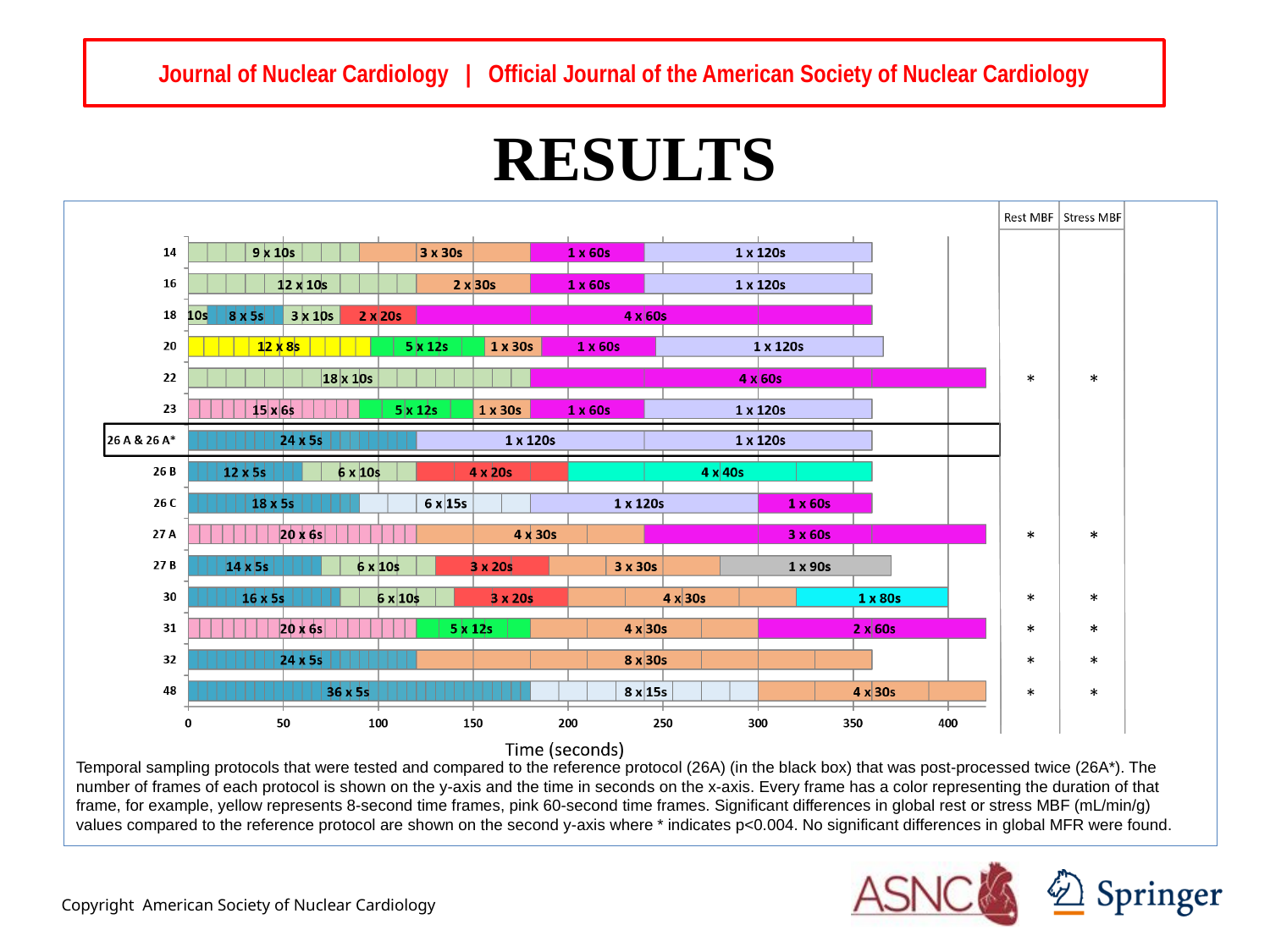

Journal of Nuclear Cardiology | Official Journal of the American Society of Nuclear Cardiology
# RESULTS
Temporal sampling protocols that were tested and compared to the reference protocol (26A) (in the black box) that was post-processed twice (26A*). The number of frames of each protocol is shown on the y-axis and the time in seconds on the x-axis. Every frame has a color representing the duration of that frame, for example, yellow represents 8-second time frames, pink 60-second time frames. Significant differences in global rest or stress MBF (mL/min/g) values compared to the reference protocol are shown on the second y-axis where * indicates p<0.004. No significant differences in global MFR were found.
Copyright American Society of Nuclear Cardiology

## Slide 5
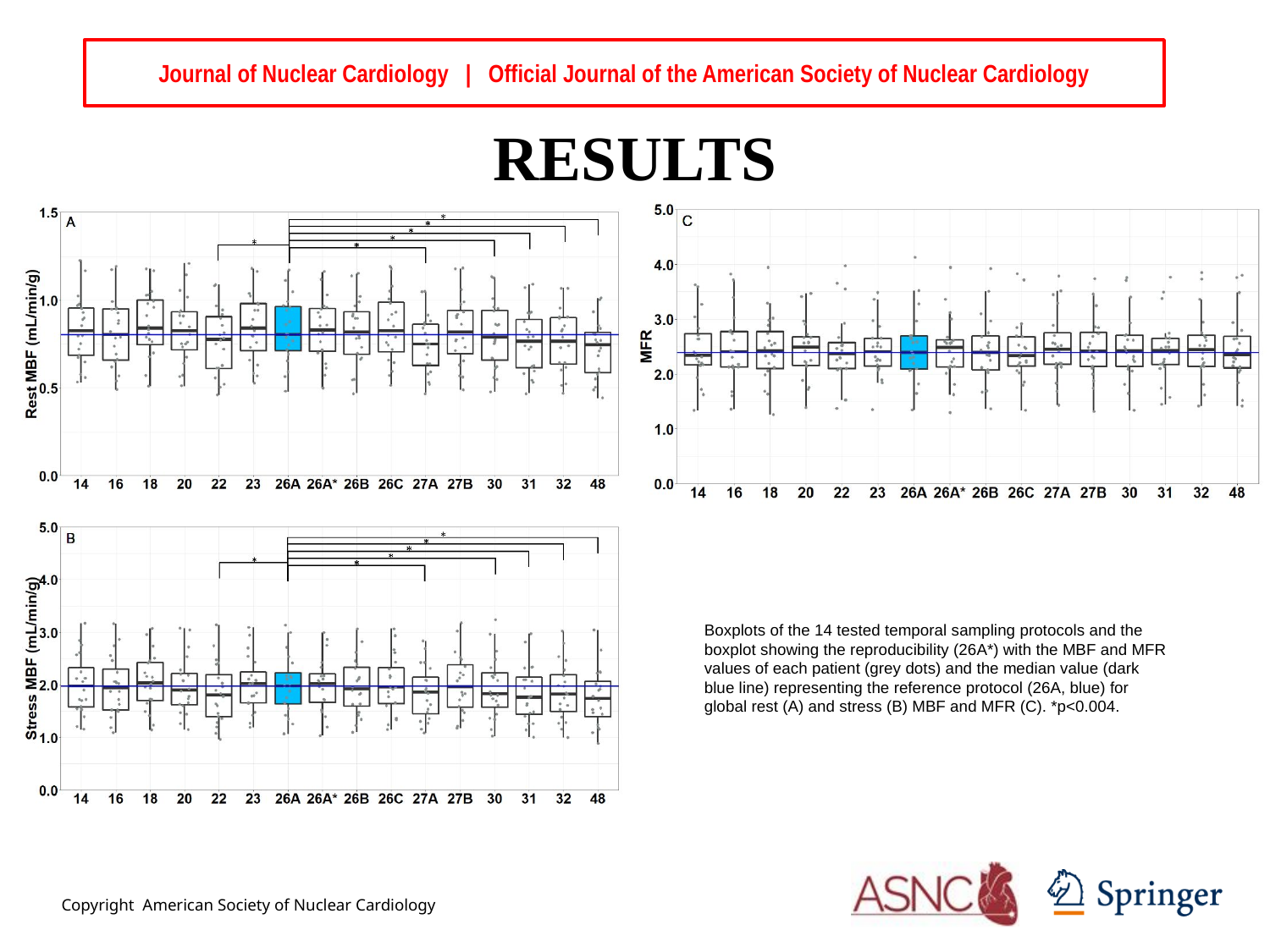

Journal of Nuclear Cardiology | Official Journal of the American Society of Nuclear Cardiology
# RESULTS
Boxplots of the 14 tested temporal sampling protocols and the boxplot showing the reproducibility (26A*) with the MBF and MFR values of each patient (grey dots) and the median value (dark blue line) representing the reference protocol (26A, blue) for global rest (A) and stress (B) MBF and MFR (C). *p<0.004.
Copyright American Society of Nuclear Cardiology

## Slide 6
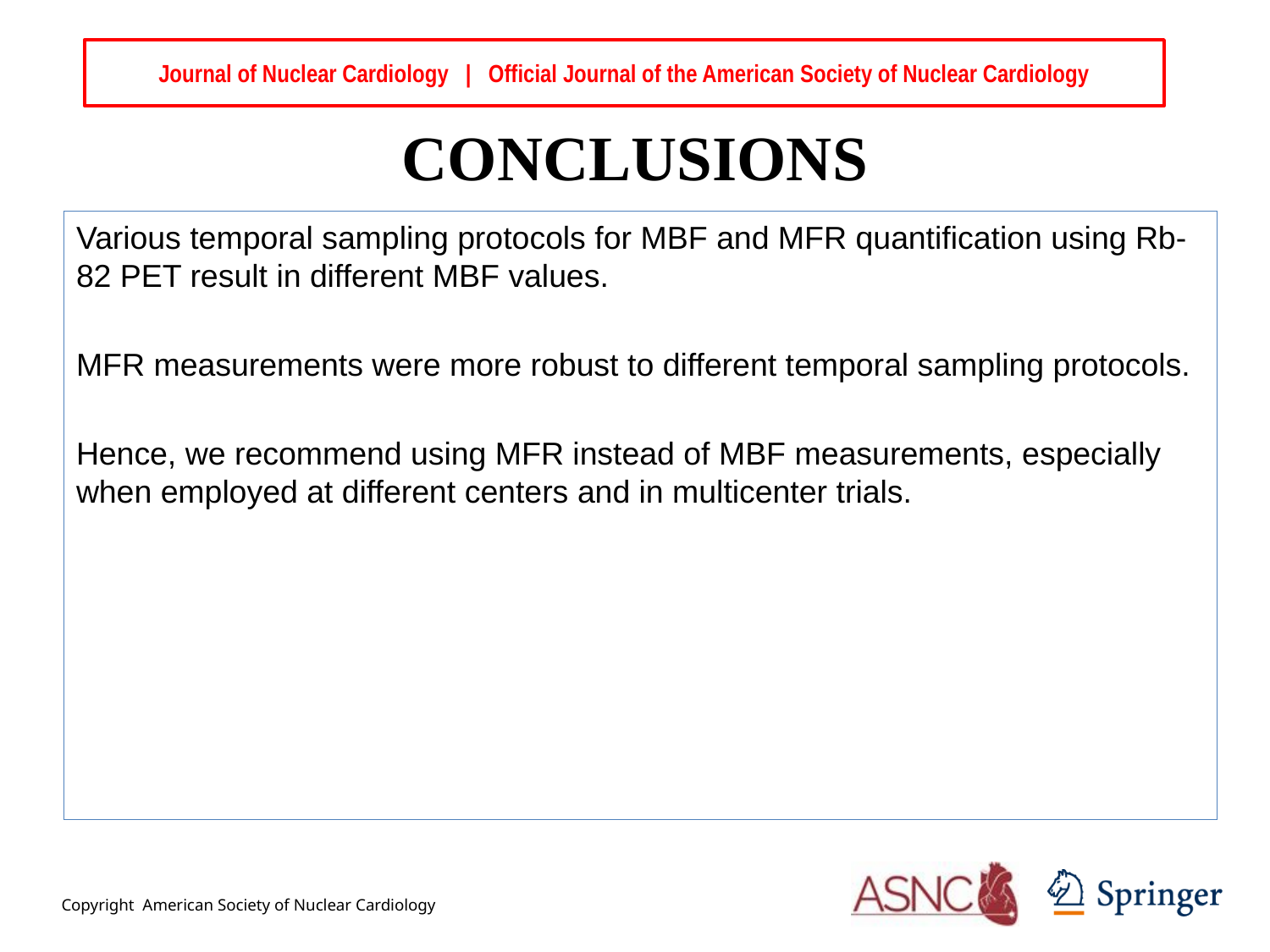

Journal of Nuclear Cardiology | Official Journal of the American Society of Nuclear Cardiology
# CONCLUSIONS
Various temporal sampling protocols for MBF and MFR quantification using Rb-82 PET result in different MBF values.
MFR measurements were more robust to different temporal sampling protocols.
Hence, we recommend using MFR instead of MBF measurements, especially when employed at different centers and in multicenter trials.
Copyright American Society of Nuclear Cardiology
